# Best Practices for Integrating Medical Students Into Telehealth Visits

**DOI:** 10.2196/27877

**Published:** 2021-04-21

**Authors:** Maria Wamsley, Laeesha Cornejo, Irina Kryzhanovskaya, Brian W Lin, Joseph Sullivan, Jordan Yoder, Tali Ziv

**Affiliations:** 1 Department of Medicine University of California San Francisco School of Medicine San Francisco, CA United States; 2 University of California San Francisco School of Medicine San Francisco, CA United States; 3 Department of Emergency Medicine University of California San Francisco School of Medicine San Francisco, CA United States; 4 Department of Emergency Medicine Kaiser Permanente Northern California San Francisco, CA United States; 5 Department of Neurology University of California San Francisco School of Medicine San Francisco, CA United States; 6 Kaiser Permanente Northern California Oakland, CA United States

**Keywords:** telehealth, undergraduate medical education, workplace learning, ambulatory care, telehealth competencies, medical education, student education, digital learning, online learning, ambulatory, digital health

## Abstract

Telehealth has become an increasingly important part of health care delivery, with a dramatic rise in telehealth visits during the COVID-19 pandemic. Telehealth visits will continue to be a part of care delivery after the pandemic subsides, and it is important that medical students receive training in telehealth skills to meet emerging telehealth competencies. This paper describes strategies for successfully integrating medical students into telehealth visits in the ambulatory setting based on existing literature and the extensive experience of the authors teaching and learning in the telehealth environment.

## Introduction

*Telehealth* and *telemedicine* are terms that are used interchangeably in much of the existing literature and have become an increasingly important part of health care in the last several decades [1]. Telehealth or virtual visits are defined as live, synchronous, interactive encounters between a patient and a health care provider through video, telephone, or live chat [2]. The COVID-19 pandemic has greatly accelerated the use of telehealth visits to improve patient access to care and minimize risks to patients and health care providers [3,4], and even after the pandemic subsides, telehealth will remain an important and growing modality for care delivery [5]. The benefits of telehealth include increased access to care in remote or rural areas, mitigation of workforce shortages, improved chronic disease management, and improved health outcomes [6].

Given the rise in telehealth, it is important to prepare medical students for effective participation in telehealth visits and share best practices for integrating students into telehealth visits. The 2015-2016 Liaison Committee on Medical Education Annual Medical School Questionnaire reported that over a quarter of US medical schools have implemented telehealth training in their preclinical curriculum and nearly half have implemented it in their clinical curriculum [7]. However, much of the existing literature on telehealth for learners focuses on residents [3,8,9] or telehealth curricular descriptions for medical students [7,10-13]. There is limited but growing literature on integrating medical students into telehealth visits [14-17].

Integrating students into telehealth visits has several potential advantages. It can overcome space limitations in clinical settings, provide opportunities for learners to participate in clinical care at a distance including in settings that may be less accessible (eg, international or rural settings), provide a window into patients’ home environments, and allow students to participate in specialty consultations and interprofessional care. Telehealth provides teachers with opportunities both for direct observation of students’ patient communication and physical examination skills and for teaching focused on clinical reasoning [18].

In this paper, we describe strategies for effectively integrating medical students into telehealth visits in the ambulatory setting. We derive our approach from the educational literature and from our extensive experience with direct recommendations for teachers who have integrated or wish to integrate learners into telehealth visits. We have organized our suggestions based on Billett’s framework of workplace learning that highlights the importance of pedagogic practices before, during, and after practice-based experiences [19]. We do acknowledge that there is overlap between these categories; however, we feel that it is helpful for teachers to have a framework for approaching the incorporation of learners into telehealth experiences.

## Before the Visit

### Know Your Telehealth Platform

Facility with your telehealth platform is imperative to maximize time focused on teaching, observation, and learner assessment. Institutional decisions to use encrypted software packages approved for the transmission of protected health information leave health care providers working within the confines of systems and software packages that may be unfamiliar. Institutional training and online tutorials can enhance comfort and skills with specific technology platforms and increase familiarity with system features that may be disabled, enabled, or customized. To build patient trust and facilitate the teaching experience with the learner, it is critical that the teacher is comfortable with the telehealth platform and can troubleshoot challenges in advance of any telehealth visit.

### Provide Students With a Telehealth Curriculum That Is Aligned With Telehealth Competencies

The literature describes various telehealth curricula for medical students [7,10,11,13] and residents [3,8,9,20-22]. There has been an attempt to define both general [23] and discipline-specific telehealth competencies at the postgraduate level [8]. The American Association of Medical Colleges recently released telehealth competencies for the recent medical school graduate entering residency, the graduating resident entering practice, and the experienced practicing physician [24]. It is important to note that teachers may not yet have mastered telehealth competencies given the evolving nature of these competencies and the relatively recent expansion of telehealth.

Based on the available literature, telehealth curricula should include the following key content areas: technical skills needed to operate equipment and software, including troubleshooting difficulties; professionalism in telehealth, including review of informed consent and patient privacy; telehealth communication skills [25-27]; physical examination skills in the telehealth environment [28,29]; and affordances and limitations of telehealth visits, including the potential for telehealth to increase disparities in care [30]. It is important to specifically teach and coach students through sensitive aspects of patient history, which can include eliciting social and mental health histories and intimate partner violence screening [31] and which may be more challenging in the virtual environment. Strategies such as ensuring safety and privacy prior to initiating a telehealth visit by asking simple yes-or-no questions can be taught and modeled for learners, who in turn can be provided with opportunities to practice these skills through roleplay or simulation. Described methods for delivery of telehealth curricula include e-learning, lectures, and small group discussions. In addition, it is essential to provide opportunities for telehealth skills practice with feedback, which can be accomplished using objective structured clinical examinations in a simulated telehealth environment or in the context of patient care [8,10,32].

### Prepare Students for Success in the Virtual Visit

The first step to student success in a virtual visit is ensuring access to the appropriate technology and a private space in which to successfully conduct a telehealth visit. There may be disparities in student access to reliable broadband service, and many students may live in shared living spaces without adequate access to the private space needed for a telehealth visit. Medical schools can address these disparities by providing private rooms with Wi-Fi access for students to conduct telehealth visits or through providing Wi-Fi hotspots for learners without access to reliable broadband service.

To contribute to the team and learn most effectively, students require orientation and goal setting at the start of each telehealth session. As Knowles’ theory of andragogy outlines, adult learners are self-directed and learn best when engaged in the workplace with authentic roles [33]. In addition, learners involved in formulating task strategies perform better with a higher self-efficacy than those who do not participate in formulating strategies [34]. Establishing a sense of the student’s level of medical knowledge and any other core skills required as well as considering the student’s prior experience with telehealth will prepare for a more productive session. Choose patients together from the schedule with the highest learning potential and match the patients’ demographics, chief complaints, problem list, and presentation complexity with the student’s learning goals for the session. Share with the student your history of caring for the patient and any tips or pearls from previous encounters that will help the student. For example, if you know the patient tends to prefer a certain approach, this is helpful to share with the student in advance. Prime the student for success by starting with easier initial tasks. Be specific with expectations by, for example, delineating the amount of time the student should spend on the visit before looping you in again, what tasks the student should accomplish, and how to best communicate with you during the visit.

### Leverage Students’ Knowledge of Technology

Ramping up to provide virtual care is challenging and may be more difficult for those clinicians who are accustomed to a particular structure and rhythm of in-person clinical visits. In contrast, students are still developing their frameworks for the clinical visit and may be more flexible in their approach. Learners may appreciate the early integration of technology in their learning [35], recognizing the significant role that telehealth will play in future outpatient care delivery. Millennials, defined as those individuals born between 1981 and 2000, currently constitute the majority of medical students. They have been shaped by a profound expansion of information technology, and their facility with various hardware, platforms, and apps can be time-saving in the telehealth work environment. Millennial learners embrace collaboration, and they thrive in flat rather than pyramidal structures [36]. Inviting a student to share with the team what they know about how to best use technology in the context of telehealth visits will strengthen the learning climate and invite collaborative learning. As Generation Z students (those born between 1997 and 2012) soon emerge among our trainees, an even more technology-focused generation will challenge us to once again rethink our relationship to technology and the ways educational practice in telehealth will necessarily evolve.

### Address Disparities in Telehealth Utilization

Although the use of telehealth visits has increased dramatically, this trend has been disproportionately generated by young, non-Hispanic White patients. Patients over 65 years, those whose primary language is not English, and those insured by Medicare or Medicaid all saw a decrease in health care utilization when practices shifted from in-person to virtual care in the context of the COVID-19 pandemic [30]. As medical training and health care delivery become more virtual, medical students will have an increasingly important role in addressing disparities in access to health care. Teachers can encourage their students to engage in local efforts to improve broadband and mobile device access in underserved communities and to challenge health system barriers to telehealth access. Support for this work can be provided through systems-improvement projects or elective courses. For example, students can reach out to those most in need of connection, such as more frail older adults, screen them for mood disorders, and connect them with community resources. In the clinical setting, teachers should prompt students to proactively reach out to patients whose primary language is not English to check if they need assistance accessing virtual care by ensuring adequate access to interpreters in the visit, screening patients for privacy or technical barriers, and teaching patients how to use the telehealth platform before the appointment begins. Addressing disparities in telehealth access is also crucial for learner development. Medical trainees’ preparedness to deliver cross-cultural care often trails other clinical milestones [37], but exposure to and discussion of health care disparities in medical school has been linked to improvements in that preparedness [38]. Development of formal curricula to adapt cultural humility and antiracism training for virtual care and role modeling these within the virtual workplace will both be imperative in this new era of medical education.

## During the Visit

### Establish and Model “Webside Manner”

Connection means more than just establishing a video or telephone connection between you, the patient, and the student. Most in-person visits to a medical provider involve some physical interaction, such as a handshake or other nonverbal interaction. Virtual visits have changed that dynamic, requiring new strategies for “webside manner” to establish this extremely important personal connection. To address these challenges in communication and opportunities for relationship building, the authors of the Stanford Presence 5 [26] adapted their original evidence-based practices to help clinicians foster humanism during clinical encounters for telehealth visits [26]. Body positioning, eye contact, nodding, smiling to demonstrate a listening posture, and allowing extra pauses before speaking to account for lag time are particularly important when engaging in a telehealth visit, especially with three or more participants (patient, teacher, and student) [39]. To allow for optimal visualization of body language, position the camera in a way that allows others to see your torso and arms and use gestures as you would in person but keep the gestures in the square of your body (ie, closer to your shoulder). Be mindful that gestures may appear more unnatural with virtual backgrounds. Although it is natural to look at participants’ faces and video on the screen, from the perspective of the student or patient, this does not come across as direct eye contact. Try to maintain direct eye contact by looking at the camera when you are speaking. Consider putting something next to the camera lens that will remind you to focus your gaze there and close other computer windows to minimize distractions.

### Adapt the Students’ Authentic Roles To Be Commensurate With Their Clinical Developmental Stage

To establish an inviting virtual learning climate, you can start to iteratively develop the student’s telehealth skills. Telehealth provides a rich opportunity for the student to join the greater community of practice [40], as they quickly learn competencies ranging from medical knowledge to systems-based practice. Consider the gradient of competency in the many domains within telehealth just as you would a continuum of competency in medical knowledge. Eventually the student might participate in all aspects of telehealth, including preparing for the session with appropriate technology in place, precharting, obtaining a history, completing a virtual physical exam, and providing patient education with anticipatory guidance. However, these steps might be considered building blocks and can be approached stepwise with supported participation matching the student’s skill development [41]. Initially, students might listen in by telephone or video with the teacher and patient. As familiarity with the basic technical tools required to conduct a visit grows, the student can engage in independent communication with the patient for a portion of the encounter. One helpful framework to consider the student’s skills and developmental progress is the Reporter, Interpreter, Manager, Educator framework [42], which describes the progression of student skills during the clinical clerkship year. For students in the reporter stage, eliciting an initial history in the telehealth visit might be an appropriate task, while students in the manager stage might be able to wrap up the visit with the patient, communicating the plan and next steps with faculty supervision. These expectations should be discussed with the learner as part of the previsit preparation. Students may also follow up on results of laboratory examinations and studies after the encounter. Frequent feedback and flexibility [43] to gauge competency while cultivating a growth mindset will allow the student to progress across multiple competencies.

### Ensure “Sidelines Communication”

It is worth recognizing that when students and teachers work together in person, they have frequent points of contact. It is therefore important during telehealth sessions to preserve and promote opportunities for the student and teacher to communicate throughout the session. Dialogue is an important tool for building trust between students and teachers and can shift the power dynamic to provide students with a sense of expertise and autonomy [44]. The day-to-day interactions between students and teachers give teachers the opportunity to shape and understand the identity and roles of the medical students and provide students the dynamic to develop their professional identity [45]. It is therefore not surprising that medical students highly value having teachers who are readily available [44]. Agree on a private and reliable messaging platform for “sidelines communication” during the clinic session to communicate about timing, address questions, or provide support. Options include secure text messaging, computer communication platforms, phone calls, and videoconferencing chat functions. Which sidelines communication method you choose should be dictated by institutional privacy guidelines to comply with regulations such as the HIPAA (Health Insurance Portability and Accountability Act), institutional technical fire walls, and personal preference. Regardless of which sidelines communication you use, establishing a clear communication workflow allows students greater independence and teachers more efficient use of time while maintaining appropriate supervision.

### Foster Relationships: Engage the Patient and the Student Within the Virtual Environment

Telehealth visits provide both additional challenges and opportunities for provider–patient communication and relationship building between the student, teacher, and patient. When reflecting on telehealth visits, patients report difficulty finding opportunities to speak, a sense that providers pay less attention to them, and an inability to establish a connection with their provider [46]. However, telehealth visits also present an opportunity to engage with patients in their own environments. At the beginning of the telehealth visit, extra consideration should be given to student introductions and consent, as creating a connection can be more difficult virtually. Best practices for in-person interactions, such as exploration of emotional cues, use of open-ended questions, and the teach-back method [47], should be adapted, modeled, and encouraged during virtual interactions. Similarly, relationship-centered care can also still be achieved through telehealth visits [48]. After first ensuring safety, privacy, and appropriate occasion, engaging in the patient, student, or provider’s home environments can allow for a greater connection. Family members previously unable to attend office appointments can be involved, while pets or other important facets of patients’ lives can add depth to the interaction. Lastly, be aware of virtual meeting fatigue that results from not having full access to nonverbal cues and the mental fatigue that results from having to process the accumulation of these important missing elements of in-person social interaction [49]. Engaging in relationship building and focusing on mindful communication can help prevent burnout within more virtual clinical and teaching environments [50].

### Build In Opportunities To Teach and Observe the Virtual Physical Exam

Eliciting student learning goals in advance of the session will provide you with a focus for your teaching and observation. If the student identifies questions about how to perform an exam maneuver in your previsit conversation, take time to consider together how to most effectively perform the exam during the encounter. Even the more challenging maneuvers can be conducted over video or telephone. Plan in advance which specific examination maneuvers the student will perform and how to communicate the instructions for the exam to the patient. Consider with the student what can be gleaned from the encounter: how the patient tells their story; aspects of the history that help build the differential diagnosis; which data are available from wearable devices; or what findings can result from the “Telehealth Ten,” a patient-assisted clinical examination to help guide providers in their physical examination and clinical reasoning over telemedicine [28]. Clerkship students have noted that they appreciate timely feedback during the telehealth encounter or right after the visit, and virtual exam skills observation allows for a focused discussion around behaviors the student can keep, start, or stop doing [14]. During the visit, it may become clear that the symptoms discussed or signs noted in the virtual examination require transition to an in-person visit. Preparing the student for this possibility by explicitly exploring it with the student in advance and discussing what options exist for transitioning patients to in-person visits will allow the student to consider the opportunities and limitations of the telehealth encounter. Additionally, modeling for the student how to approach this conversation with the patient provides another opportunity for student learning.

## After the Visit

### Ensure a Post-Session Huddle: Set Aside Time To Debrief and Give Feedback

As the closing guidepost on the telehealth journey with the student, a postsession huddle creates the opportunity to reflect on the visit, share feedback with the student based on their identified goals, and create a plan or a Specific, Measurable, Attainable, Realistic, and Time-Bound (SMART) goal [51] for the next telehealth visit and the time between visits. Whether you are working with a student for two sessions or twenty-two sessions, providing feedback in the moment based on your observations can help to stimulate the student’s growth mindset and enhance the telehealth learning experience [52]. Having a feedback dialogue in a postsession online huddle without the patient present allows the teacher to share reinforcing and redirecting feedback based on their observations [14]. One potential structure for the postsession huddle is the “ask-tell-ask framework” [53]. First, ask the student to reflect on their own performance and then share (tell) your specific observations and feedback on the student’s performance and developing telehealth skills. You can share your screen in the virtual platform to highlight any particular observations. After sharing your thoughts, ask the student for their reaction to your feedback. Close the postsession huddle with a request to the student to generate a SMART goal or a plan about how to continue progressing in their telehealth skills development. Students may share their SMART goal via electronic messaging or through a shared platform for tracking student goals and progress.

### Engage in Virtual Care Across Specialties

When patients are seen by other health care providers, either in a different specialty or profession, it is often difficult for students to participate in-person if these appointments occur in different locations. Telehealth provides an opportunity for students to more readily join visits across departments with health care providers of diverse specialties and professions, enhancing opportunities for building longitudinal relationships with patients. Through active participation in patient care in different settings, the student can bridge health care providers across specialties and benefit from experiential learning, constructing knowledge and meaning from an authentic experience [41]. Encourage the student to solicit patient and provider permission in advance of joining a visit virtually. Students can provide additional support, navigation, education, and advocacy for patients during virtual visits in different settings [54]. With knowledge of the patient’s history and diagnoses, students can share additional background and context for the health care provider. Encourage students to check for patient understanding before, during, and after the visit, as this is particularly important in helping the patient navigate multiple settings and ensuring that the patient and family members have an understanding of the impression and plan from every visit; furthermore, it provides the student with a better perspective of the patient’s care experience.

## Conclusions

It is clear that telehealth visits will continue to be an expanding modality for the provision of care in the future. Consequently, medical students will need to be trained to meet telehealth competencies, and teachers will need to be able to coach medical students in these important skills. Creating opportunities for students to engage in telehealth visits using the above outlined best practices will provide them with opportunities to practice telehealth skills safely and effectively with guidance and feedback from prepared teachers.

## Abbreviations

HIPAA: Health Insurance Portability and Accountability Act
SMART: Specific, Measurable, Attainable, Realistic, and Time-Bound
